# Mathematical modelling of oxygen gradients in stem cell-derived liver tissue

**DOI:** 10.1371/journal.pone.0244070

**Published:** 2021-02-08

**Authors:** Joseph A. Leedale, Baltasar Lucendo-Villarin, Jose Meseguer-Ripolles, Alvile Kasarinaite, Steven D. Webb, David C. Hay

**Affiliations:** 1 Department of Mathematical Sciences, University of Liverpool, Liverpool, United Kingdom; 2 MRC Centre for Regenerative Medicine, University of Edinburgh, Edinburgh, United Kingdom; 3 Department of Applied Mathematics, Liverpool John Moores University, Liverpool, United Kingdom; University of Tampere, FINLAND

## Abstract

A major bottleneck in the study of human liver physiology is the provision of stable liver tissue in sufficient quantity. As a result, current approaches to modelling human drug efficacy and toxicity rely heavily on immortalized human and animal cell lines. These models are informative but do possess significant drawbacks. To address the issues presented by those models, researchers have turned to pluripotent stem cells (PSCs). PSCs can be generated from defined genetic backgrounds, are scalable, and capable of differentiation to all the cell types found in the human body, representing an attractive source of somatic cells for *in vitro* and *in vivo* endeavours. Although unlimited numbers of somatic cell types can be generated *in vitro*, their maturation still remains problematic. In order to develop high fidelity PSC-derived liver tissue, it is necessary to better understand the cell microenvironment *in vitro* including key elements of liver physiology. *In vivo* a major driver of zonated liver function is the oxygen gradient that exists from periportal to pericentral regions. In this paper, we demonstrate how cell culture conditions for PSC-derived liver sphere systems can be optimised to recapitulate physiologically relevant oxygen gradients by using mathematical modelling. The mathematical model incorporates some often-understated features and mechanisms of traditional spheroid systems such as cell-specific oxygen uptake, media volume, spheroid size, and well dimensions that can lead to a spatially heterogeneous distribution of oxygen. This mathematical modelling approach allows for the calibration and identification of culture conditions required to generate physiologically realistic function within the microtissue through recapitulation of the *in vivo* microenvironment.

## 1 Introduction

The optimisation of the *in vitro* niche for cell culture and tissue engineering is critically important [1]. Cell culture protocols are becoming increasingly scrutinised to determine if the reported methodologies deliver experimental consistency and reproducibility [2]. This is an important consideration as irreproducibility undermines the validity and utility of the *in vitro* model when extrapolating to human physiology.

It is often the case in cell culture that *in vitro* data are used to infer properties about the cells of interest that can be translated into understanding of the system *in vivo* [3]. In order to assert such extrapolative interpretations, one must fully acknowledge and account for the intrinsic differences between *in vitro* and *in vivo* environments. Important *in vitro* factors to consider include whether the cells are arranged in 2D or 3D, and the effects of the local microenvironment. The supply of nutrients such as oxygen can be more easily controlled for 2D cell culture but the use of more physiologically relevant 3D cultures results in spatially varying nutrient gradients [4]. Therefore the delivery of functional and phenotypically stable liver tissue requires precise control of the size of 3D liver spheroids [5,6]. It can be difficult, and costly, to investigate the impact of cell culture protocol on the establishment of 3D nutrient gradients and thus it can be a somewhat overlooked feature when preparing optimised experimental conditions.

Mathematical models and *in silico* simulations can provide estimates of difficult-to-measure system properties, such as oxygen gradients, by describing system processes and mechanisms explicitly and performing virtual experiments computationally. This methodology allows the researcher to investigate and optimise various cell culture conditions in order to determine relevant cell culture protocols as well as gaining a deeper mechanistic insight into the system. This enhanced mechanistic understanding can assist the researcher when interpreting experimental data acquired and how it relates to fundamental properties of the cells as well as speculations on *in vivo* extrapolation.

The generation of human tissues from renewable sources of somatic cells with a defined genetic background has enormous potential for modern medicine [7]. However, these processes require optimised cell culture to ensure the delivery of unlimited quantities of human cells and tissues at large scales. Current sources from which liver cells can be obtained include primary adult human hepatocytes, hepatic progenitor cells, cancer cell lines and animal hepatocytes. While these cell sources are enabling, they also possess some drawbacks, which limit their routine use. These drawbacks include incomplete hepatocyte phenotype, genomic instability, variable function and species differences [8]. PSCs represent a source of cells that can give rise to all somatic cell types found in the human body with self-renewal and differentiation properties that make them the ideal candidate to cope with the current demands of liver models [9]. The employment of mathematical modelling to optimise PSC-derived liver tissue may result in improved current culture conditions that can recapitulate liver biology more faithfully and improve the likelihood of technology translation.

The methodology described in this article and used to build the *in silico* framework herein builds upon previous work, primarily that of Leedale et al. [10]. The application of mathematical modelling for describing oxygen gradients within cellular spheroids has a relatively rich body of literature from which to build upon [11–16]. These studies originally focused largely on the emergence of hypoxia within tumour spheroids but have since expanded to study the spatiotemporal dynamics of many environmental signals within 3D cellular systems. The methodology presented here details how specific properties of the microenvironment such as: well-geometry; media volume; size of cell-structure; cell position; and oxygen gradients impact on PSC-derived liver spheres. This methodology should be considered appropriate for any researcher working within cell culture whose aim is to improve the relevance of their experiments via mechanistic analysis and understanding. The methodology described provides a relatively quick, transparent and economical way to determine if prolonged, complex and expensive experiments are physiologically relevant.

## 2 Materials and methods

### 2.1 Governing equations

The mathematical model describing the spatiotemporal dynamics of oxygen in cell culture is governed by the following partial differential equation,

$$\frac{\partial C}{\partial t}=D_{sph}\nabla^{2}C-\frac{V_{max}C}{C+K_{m}},$$ (1)

which estimates intracellular oxygen concentration, *C*, in mol/m^3^. This equation assumes that intra-spheroidal oxygen dynamics are governed by diffusion and consumption processes only. The intra-spheroidal diffusion rate is given by *D*_*shp*_ (m^2^/s) and oxygen metabolism assumes Michaelis-Menten kinetics with maximal oxygen consumption rate *V*_*max*_ (mol/m^3^/s) and Michaelis constant *K*_*m*_ (mol/m^3^). Oxygen dynamics within the media surrounding the cellular spheroids are assumed to be governed by diffusion only, i.e.,

$$\frac{\partial C}{\partial t}=D_{med}\nabla^{2}C,$$ (2)

where *D*_*med*_ is the diffusion rate (m^2^/s) of oxygen within the media. The mathematical model is inherently an abstract representation of the *in vitro* environment and as such, some simplifying assumptions are made. These include the assumption that cell density is uniform throughout the spheroid such that local oxygen consumption is only a property of position and oxygen concentration (i.e., *V*_*max*_ is a constant, independent of space) and that the entire spheroid consists of cells such that there are no necrotic cores of empty, non-respiring space.

### 2.2 Model geometry

**Fig 1:**
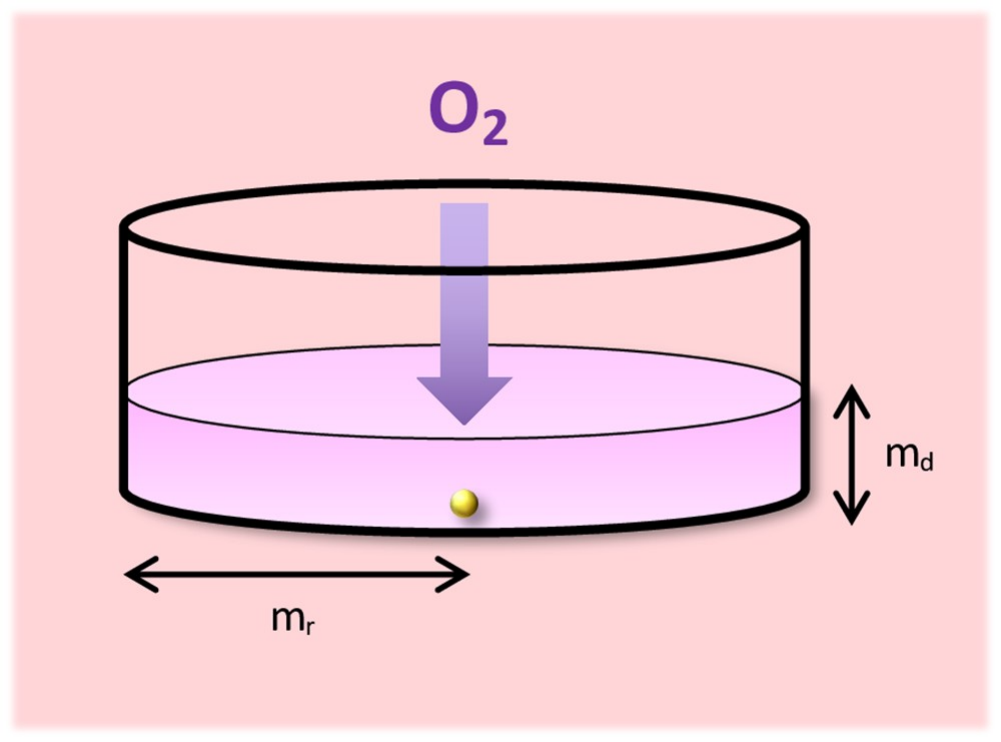
Model geometry. Model schematic for a single spheroid within an individual well of a Corning Costar 6-well plate. Well/media radius = m_r_ = 17.4 mm; media depth = m_d_ = 3.1541 mm. Atmospheric oxygen is supplied to the media surface and diffuses through the media.

Boundary conditions for the mathematical model in Eqs (1) and (2) are dependent on the model geometry, i.e., the shape and volume of media and the source of oxygen. A Corning Costar 6-well plate is used to culture the PSC-derived liver spheres of interest. Wells within this plate are cylindrical in shape with a diameter of 34.8 mm and 3 ml of media is added [17]. Based on this information the domain for the computational model could be constructed (a cylinder of radius 17.4 mm and height 3.1541 mm). A schematic of the model geometry can be seen in Fig 1.

Oxygen is supplied to the well via the upper media surface from the surrounding air, and thus we assume the following boundary condition:

$$C=C_{A},$$ (3)

at the air/media interface where *C*_*A*_ represents the atmospheric oxygen concentration in a normoxic incubator of 140 mmHg (~0.181 mmol/L O_2_), assuming an incubator temperature of 37°C and approximate sea-level altitude [18]. Zero-flux boundary conditions are assumed at all other wall-surfaces of the well such that

∇C⋅n=0, (4)

where **n** is the outward-pointing unit normal vector. At the interface between the media and the liver sphere boundary continuity and equal flux is assumed such that

$$C_{sph}=C_{med},$$ (5)

and

$$D_{sph}\nabla C_{sph}=D_{med}\nabla C_{med},’$$ (6)

on the boundary δΩ where Ω represents the liver sphere domain.

### 2.3 Parameterisation

Model parameters were identified from the literature and incorporated into the model as previously described and summarised by Leedale et al. [10]. Briefly, internal and external diffusion coefficients were defined as previously for the spheroid/oxygen system [12], as was the Michaelis constant *K*_*m*_ [19]. For this novel stem cell application, oxygen consumption rates for hepatocyte-like cells differentiated from human-induced pluripotent stem cells were used to parameterise *V*_*max*_ [20]. Model parameters are summarised in the supplementary material alongside a summary of the model equations.

### 2.4 Simulation

**Fig 2:**
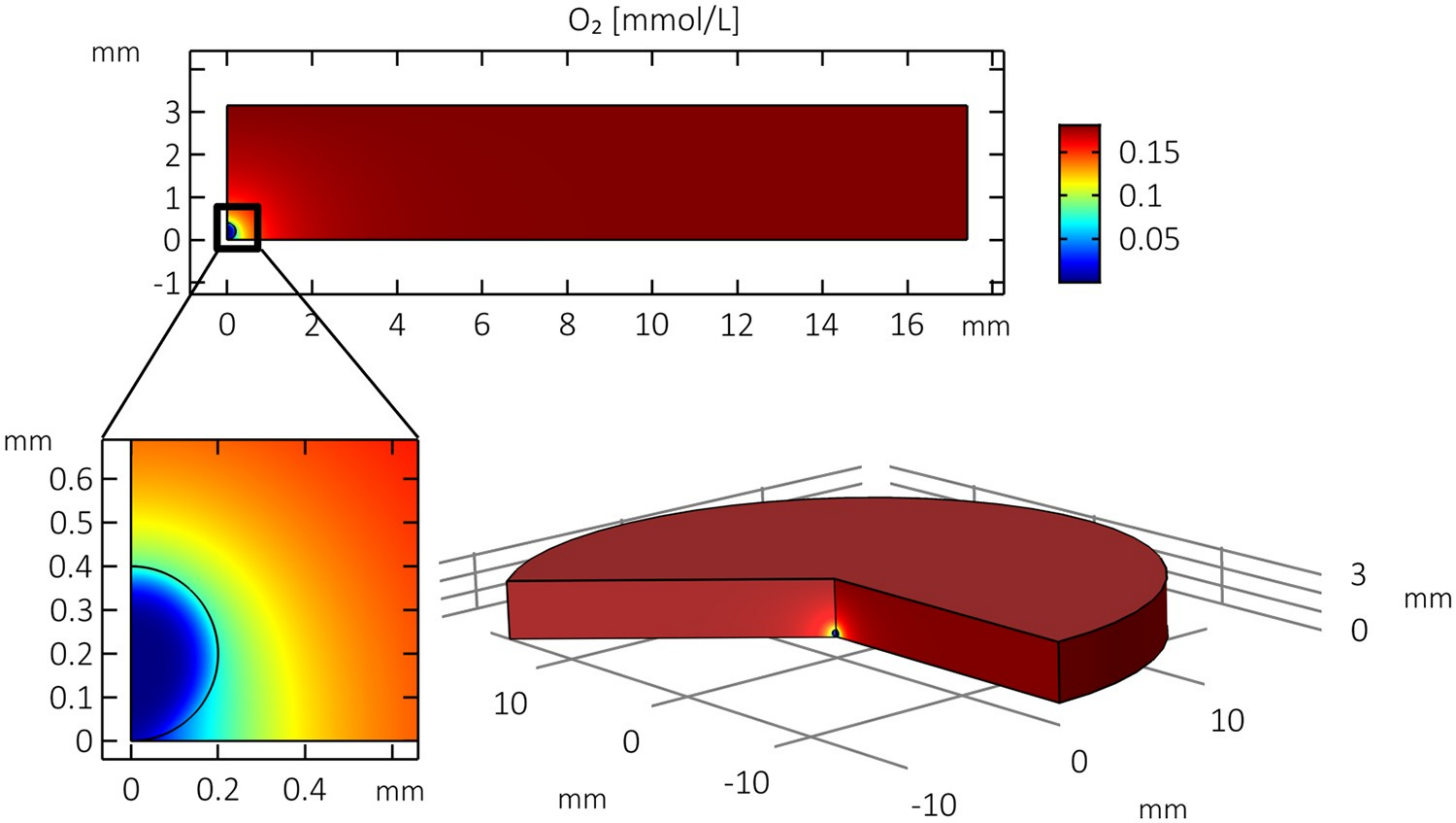
Model simulation. Illustrative 3D model simulation of oxygen distribution (mmol/L) for single spheroid (large, 200 μm radius) positioned at the bottom and centre of the well in a symmetric model.

Model simulations are performed using COMSOL Multiphysics software to determine the steady-state spatial distribution of oxygen concentration. A simplification of the mathematical model can be implemented in order to study the characteristics of a single spheroid within this system by exploiting cylindrical symmetric assumptions. For a single spheroid located along the central vertical axis of the well, we assume that the model geometry is symmetric about this “z-axis” and can be represented by a 2D plane that is rotated to visualise the 3-dimensional results. The results of an illustrative simulation of this simplified version of the model can be seen in Fig 2 showing the steady-state distribution of oxygen concentration throughout the well and spheroid. The spheroid is assumed to have a radius of 200 μm and is located at the bottom of the well. We observe that oxygen concentration is relatively uniform and close to atmospheric levels throughout most of the well. However, near to the spheroid boundary, oxygen concentration is depleted and inside the liver sphere there is less oxygen due to cellular consumption. We notice a slight radial asymmetry in the oxygen distribution as the upper portion of the spheroid is relatively better-supplied with oxygen than the lower boundary of the spheroid resting on the well bottom. This feature is described in more detail by Leedale et al. [10].

### 2.5 Optimisation

In order to maximise the *in vivo*-like relevance of hepatic spheroids cultured *in vitro*, it is desirable to replicate the oxygen gradient observed along the liver sinusoid within the spheroid [10]. The liver sinusoid is a fundamental architectural sub-unit of the liver that encompasses a range of oxygen concentrations along its length, due to the delivery of oxygenated blood from the hepatic arteriole and portal vein which flows along the sinusoid and is drained at the central vein. This gradient corresponds to a zonation within the sinusoid such that oxygen tensions range from approximately 65 mmHg (~8.5%, 0.084 mmol/L) in the periportal region (closest to the portal vein) to 35 mmHg (~4.6%, 0.045 mmol/L) in the pericentral region (closest to the central vein) [21,22]. This gradient can impact upon hepatocyte characteristics and functionality along the sinusoid and so it is important that *in vitro* testing of 3D hepatocyte culture includes these environmental properties to ensure relevance of resulting experimental data [23].

Properties of the model were analysed in order to identify optimal operating conditions that would provide the desired oxygen gradient within a single PSC-derived liver spheroid. In this study these properties included spheroid size and suspension height within the well, two features that have been observed to vary within the development and culture of these particular liver spheres (visual observation at Prof. David Hay’s laboratory, Edinburgh). This analysis involves repeated model simulations such that the features of interest are investigated via a range of suitable parameter perturbations (see supplementary Figure in S1 File for an illustrative example of oxygen distributions being affected by spheroid height). Quantification of minimum and maximum oxygen concentrations within the spheroid, as well as the average value around the spheroid boundary, are calculated and can be compared with reference values for *in vivo* periportal and pericentral liver oxygen tensions. In order to determine the optimal combination of analysed properties (in this case, spheroid radius and height within the well) that exhibit the closest representation of the *in vivo* gradient, an error function is defined such that relative differences between the simulated and reference oxygen values can be calculated:

$$Combined\;\%\;error=\frac{1}{2}\left(\frac{\left|C_{max}-C_{PV}\right|}{C_{PV}}+\frac{\left|C_{min}-C_{CV}\right|}{C_{CV}}\right)\times 100,$$ (7)

where *C*_*min*_ and *C*_*max*_ represent minimum and maximum concentrations, respectively; *C*_*PV*_ represents *in vivo* oxygen concentrations at the portal vein (0.084 mmol/L); and *C*_*CV*_ represents *in vivo* oxygen concentrations at the central vein (0.045 mmol/L). The parameter combination (e.g., particular spheroid radius and height) that minimises this function can be said to best coincide with the *in vivo* reference oxygen concentrations of interest.

### 2.6 Maintenance of human PSCs

A hiPSC line (P106) were cultured on Laminin 521 (Biolamina) coated plates in serum-free mTeSR^™^ (STEMCELL Technologies) in a humidified 37°C, 5% CO_2_ incubator as previously described [24]. Cells were passaged routinely using Gentle Cell Dissociation reagent (STEMCELL Technologies) and seeded as small colonies of cells at a dilution of 1:6 to 1:10. hPSC were cultured in an antibiotic free medium and regularly tested for mycoplasma infection.

### 2.7 Hepatic differentiation

For hepatic differentiation, hiPSCs were dissociated using Gentle Cell Dissociation reagent (STEMCELL technologies) and seeded onto pre-coated wells with Laminin 521 (BioLamina) in mTeSR1^™^ supplemented with 10 μM Y-27632 (Biotech) at a density of 40,000 cells/cm^2^. Differentiation was initiated 24 h post seeding once cell confluency reached 40% by replacing stem cell medium with endoderm differentiation medium [RPMI 1640 containing 1x B27 (Life Technologies), 100 ng/mL Activin A (Biotech) and 50 ng/mL Wnat3a (Biotech)]. The medium was changed every 24 h for 3 days. On day 4, endoderm differentiation medium was replaced with hepatic progenitor differentiation medium, and this was renewed every second day for a further 5 days. The medium consisted of knockout (KO)-DMEM (Life Technologies), Serum replacement (Life Technologies), 0.5% Glutamax (Life Technologies), 1% non-essential amino acids (Life Technologies), 0.2% 2-mercaptoethanol (Life Technologies), and 1% DMSO (Sigma). On day 9, differentiating cells were cultured in the hepatocyte maturation medium which comprised of Hepato-ZYME (Life Technologies) containing 1% Glutamax (Life Technologies), supplemented with 10 ng/ml hepatocyte growth factor (PeproTech) and 20 ng/ml oncostatin m (PeproTech) as described previously [24].

### 2.8 Production stem cell-derived hepatospheres

Following hPSC hepatic progenitor differentiation, cells were collected as single cells using TrypLE (Thermofisher). cells were counted and resuspended at a final density of 4 x 10^6^ live cells/mL in liver sphere medium consisted of William’s E media with 10% Serum replacement (ThermoFisher), 1% Glutamax and 1% penicillin- streptomycin (ThermoFisher). The cell pellet was resuspended in liver sphere medium, supplemented with 10 μM Y-27632 (Biotech), 10 ng/mL EGF (Biotech), 10 ng/mL FGF (Peptrotech), 10 ng/mL HGF (Peprotech), 20 ng/mL OSM (Peprotech) and 50 ng/mL VEGF (Biotech). 190 μL of cell suspension was dispensed in an agarose mold with 256-microwells of 400 μm using the 3D Petri Dish mould (Sigma Aldrich) as previously described [6].

### 2.9 Protein secretion

To measure alpha-fetoprotein and albumin secretion, liver spheres were maintained in supplemented liver sphere medium without SFM-Endothelial media and in the presence of 10 μM hydrocortisone 21-hemisuccinate sodium salt (HCC). Culture media was collected after 24 h and quantified using commercially available ELISA kits (Alpha Diagnostic International). Data were normalised by total protein content measured using bicinchoninic acid (BCA) assay (Thermo Fisher).

### 2.10 Cytochrome P450 activity

To measure Cyp3A and Cyp1A2 activity, 50 μM of Luciferin-PFBE substrate (Promega) or 100 μM of Luciferin-ME (Promega) were incubated with liver spheres maintained in liver sphere medium. Cytochrome P450 activity was measured 24 h later using the P450-Glo assay kit (Promega) according to manufacturer’s instructions. Data were normalised by total protein content measured using bicinchoninic acid (BCA) assay (Thermo Fisher).

### 2.11 Histological staining

Liver spheres were fixed for at least 1 h in 4% neutral buffered formalin solution (pH 7.4) at 4°C and washed twice with PBS at room temperature before embedding in agarose. Agarose-embedded liver spheres were then embedded in paraffin and sectioned at 4 μm and stained for hematoxylin and eosin. Images were taken using a Nikon Eclipse e600 microscope equipped with a Retiga 2000R camera (Q-Imaging) and Image-Pro Premier software.

## 3 Results

### 3.1 Impact of spheroid properties on oxygen distribution

**Fig 3:**
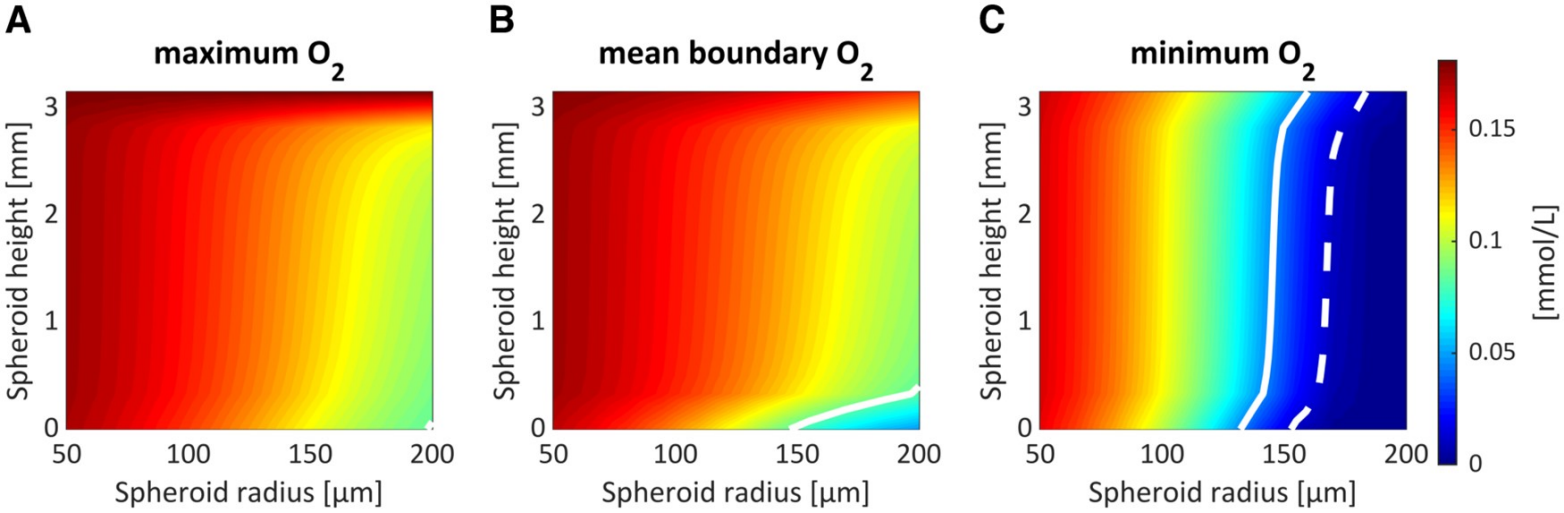
Impact of varying spheroid properties. Maximum (A), average boundary (B) and minimum (C) oxygen concentrations for a range of model parameter combinations varying spheroid radius and position (height along z-axis) within the well. Contours represent optimal *in vivo* conditions (white, solid) or hypoxia (defined as 10 mmHg, white, dashed).

Minimum, maximum and mean-boundary steady state oxygen concentrations were calculated for a range of PSC-derived liver spheres cultured within a well (Fig 3). The spheroid properties that were varied were spheroid size (radius of 50 to 200 μm) and spheroid height (range encompassing the height of the (3ml) media). Fig 3 indicates the optimal parameter pair for simulating the *in vivo* oxygen concentrations (0.084 mmol/L for maximum/mean and 0.045 mmol/L for minimum) as well as a hypoxic threshold, assumed to be 10 mmHg (0.013 mmol/L) [25]. The model suggests that, in order to exhibit approximate periportal oxygen conditions at the boundary, the *in vitro* liver spheres must be relatively large and positioned towards the bottom of the well (see white contour in Fig 3B). In order to exhibit physiologically relevant minimum values (pericentral), spheroids just need to be relatively large (see solid white contour in Fig 3C). This size varies depending on the location within the well, but ranges from a radius of approximately 130 μm at the bottom of the well to 160 μm at the top. Cells within spheroids positioned higher in the well are located nearer to the source of oxygen and so are capable of being sufficiently oxygenated at larger sizes. However, in order to avoid hypoxia, spheroids must be no larger than approximately 155 μm at the bottom and 185 μm at the top of the media (see dashed white contour in Fig 3C).

**Fig 4:**
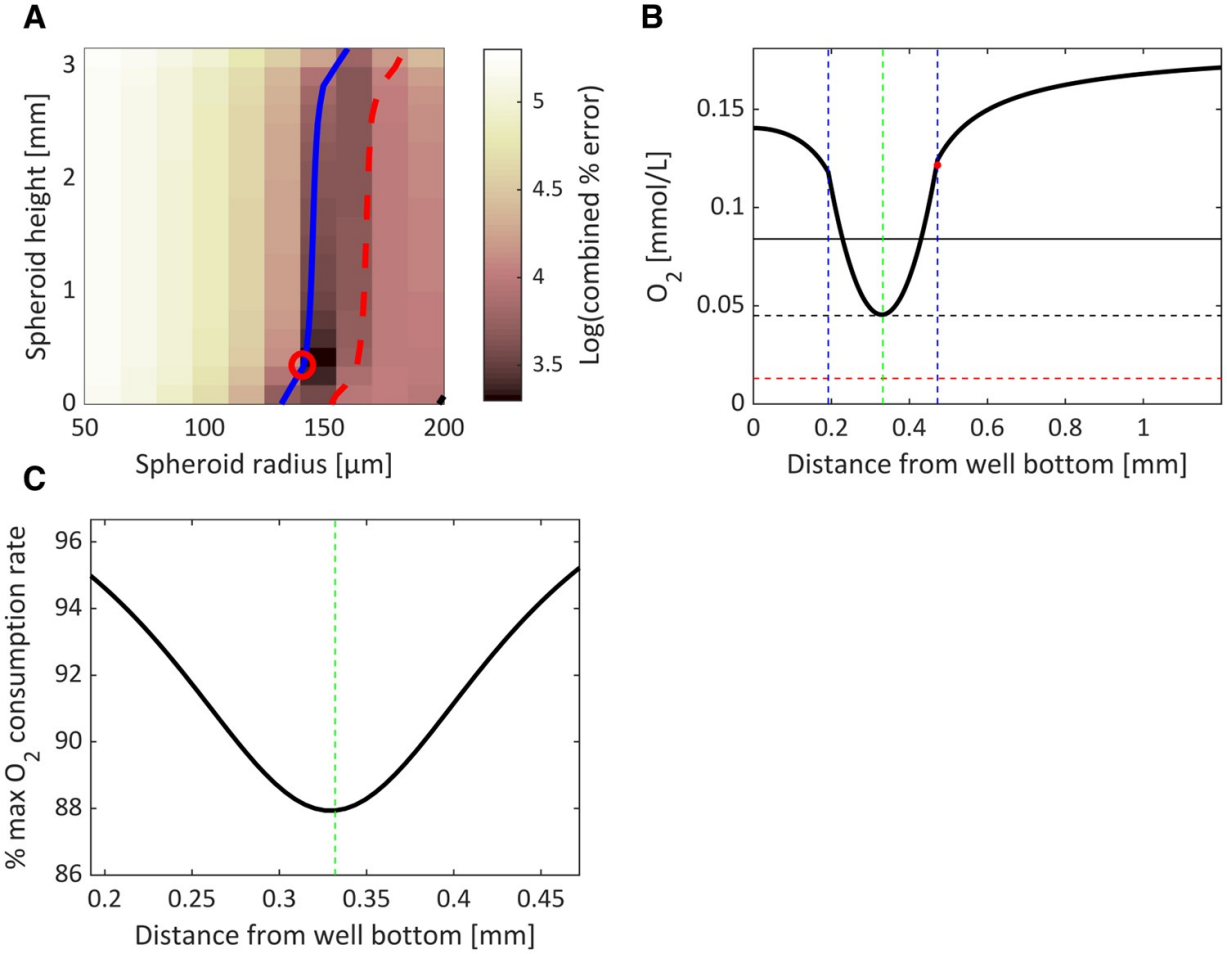
Optimising spheroid properties. Optimal model parameterisations (spheroid radius and height) are identified by calculating a combined error between model output and *in vivo* oxygen measurements (A). The minimum error (red circle) indicates the most *in-vivo*-like representation of the sinusoidal oxygen gradient. The blue contour represents parameter combinations that simulates pericentral oxygen values for the minimal spheroid concentration whereas the red contour indicates hypoxia (defined as 10 mmHg). A 1D plot is provided for the optimal model parameterisation indicating the oxygen profile along the central axis of symmetry through the well (B). The minimal value corresponds to the *in vivo* central vein value (black dashed line). The *in vivo* portal vein value (black solid line) and hypoxic threshold (red dashed line) are also indicated. The green dashed line indicates the centre of the spheroid while blue dashed lines indicate the spheroid boundary. The mean boundary concentration is represented by the red dot. The corresponding oxygen consumption rate, expressed as a percentage of the maximal rate (*V*_*max*_), is also shown for this 1D cross-section (C).

The optimal conditions providing the most physiologically relevant oxygen ranges within the PSC-derived liver spheres were determined by minimising the combined % error (given in Eq (7)) for each combination of spheroid radius and height (Fig 4A). Our analysis indicates that, for this cell type, the optimal parameter pair that minimises the combined error corresponds to a spheroid of radius 140 μm suspended 0.332 mm from the bottom of the well. A 1D representative plot through the axis of symmetry (z-axis through the centre of the well) is plotted for this optimised model parameterisation in Fig 4B. The minimal oxygen concentrations occur towards the centre of the spheroid and share the same value as those in the pericentral region of the liver sinusoid. Spheroid boundary oxygen concentrations are slightly higher than periportal regions, but this scenario prevents hypoxia and still encompasses the physiologically relevant *in vivo* range. The suspension of the spheroid above the well-bottom alleviates potential asymmetry in the oxygen profile as the oxygen supply in the surrounding media is relatively homogenous (e.g., for contrast, see asymmetric profiles for liver cell-line spheroids in Leedale et al. [10]). These oxygen levels allow for oxygen consumption rates close to the maximum value (given by *V*_*max*_) throughout the spheroid (Fig 4C).

**Fig 5:**
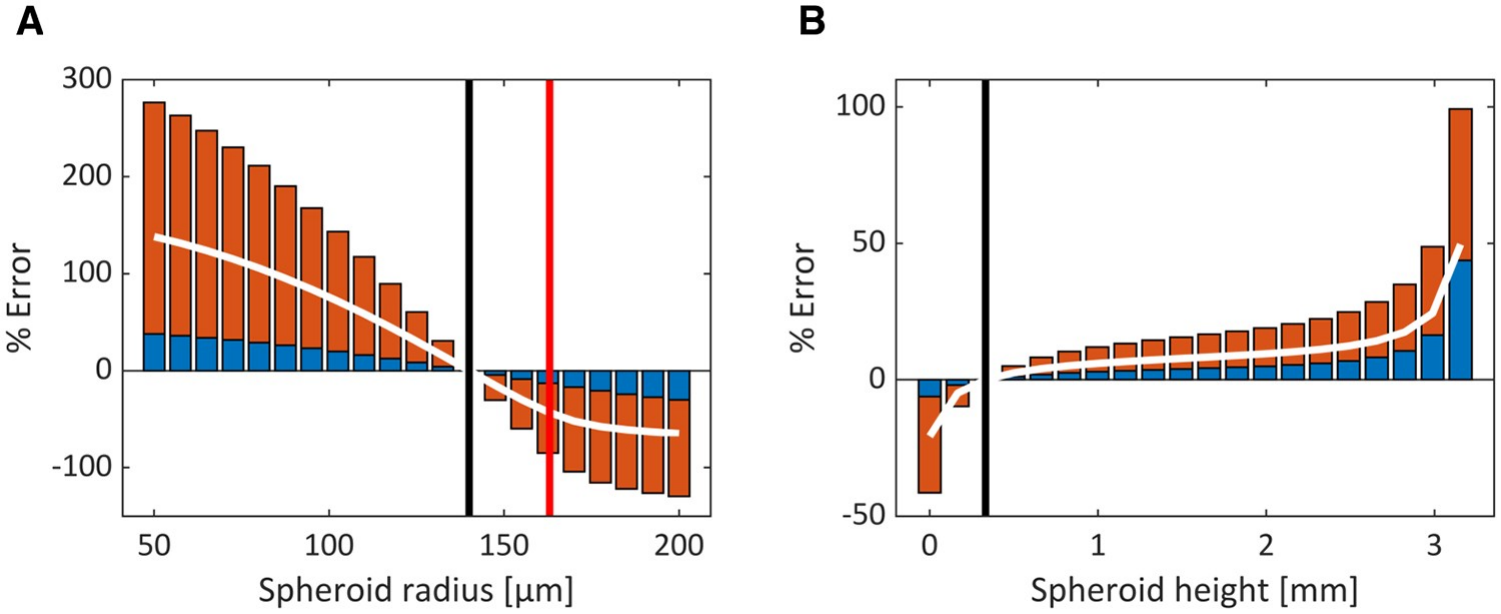
Sensitivity analysis of the optimised parameters. The % error for both minimum (red) and maximum (blue) oxygen concentrations within the spheroid are plotted for variations in spheroid radius (A) and height within the well (B). White lines indicate averaged % error; black lines indicate the optimal conditions; and the red line indicates the hypoxic threshold.

The sensitivity of the model outputs to variations in spheroid radius and height within the well can also be determined computationally (Fig 5). We observe that the spheroid radius is a relatively more sensitive parameter with a ±20% change in radius (112 to 168 μm) leading to average errors of +60 and -50% (Fig 5A). By contrast, the average errors for the spheroid suspension height within the well range from -20% to +50% for the entire range of heights from well-bottom to media surface (Fig 5B). Importantly, the model predicts that an increase in radius of just 23 μm (from 140 to 163 μm) will lead to the onset of hypoxia in the centre of the spheroid (Fig 5A).

### 3.2 Impact of multiple spheroids within a single well

**Fig 6:**
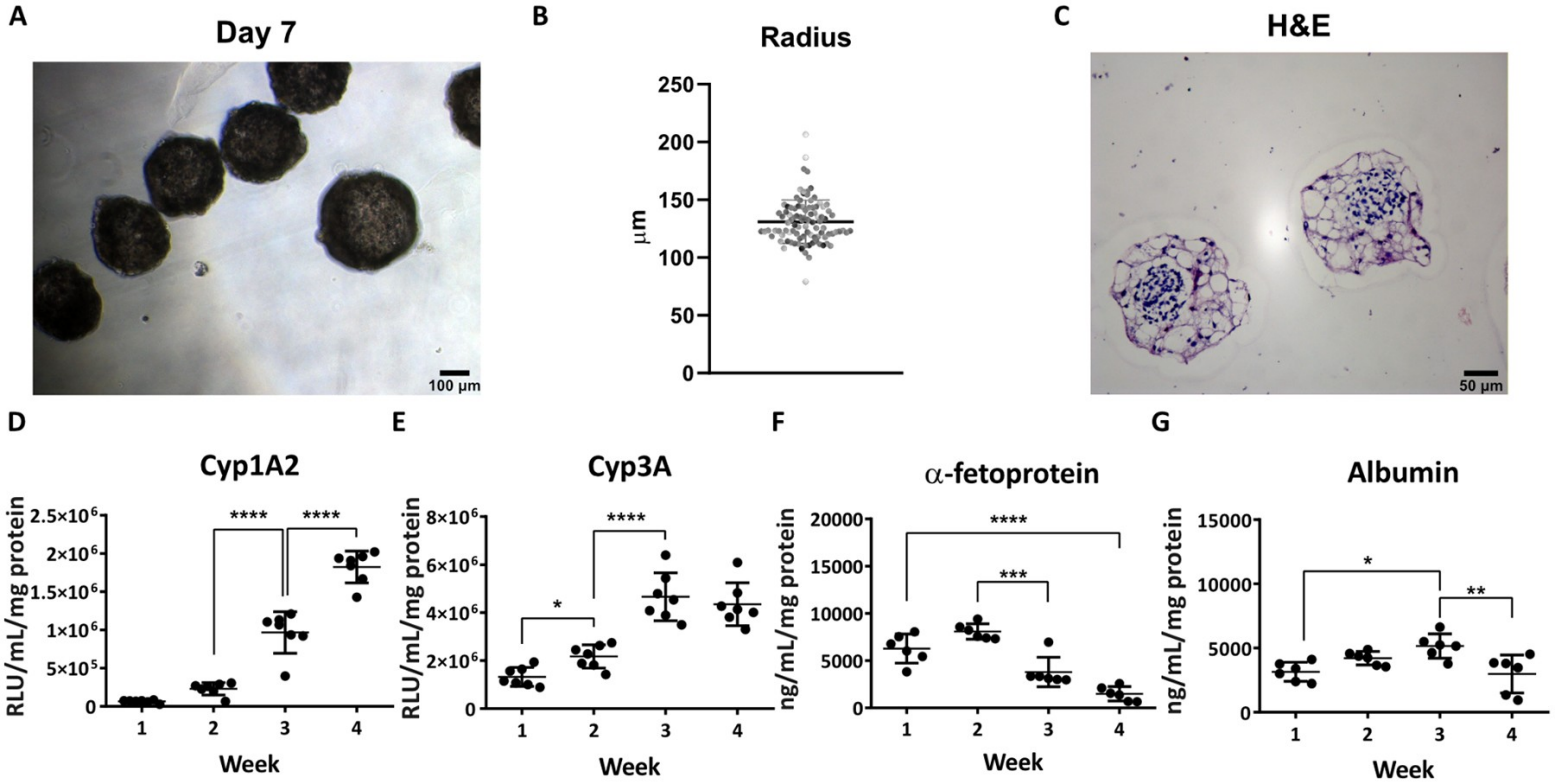
Liver sphere functional characterisation. (A) hPSC-derived spheres phase contrast image at day 7, scale bar 100 μm. (B) Radius distribution of liver spheres (mean ± SD, *n* = 100) (C) Hematoxylin and eosin (H&E) staining of hPSC-derived spheres sectioning at day 14, scale bar 50 μm. (D) Cytochrome P450 1A2 and (E) Cytochrome P450 3A activity were analysed at different time points during culture (mean ± SD, n = 7). Secretion of the serum proteins (F) alpha-fetoprotein and (G) albumin, were measured by ELISA at the denoted times (mean ± SD, n = 7). Data was analysed using the 2-way analysis of variance (ANOVA) and Turkey’s multiple comparison test (α = .05).

Stem cell derived hepatospheres were produced as previously described [26] (Fig 6A). The average sphere size was 129.72 μm (+/- 22.85 μm) (Fig 6B) and displayed non-necrotic centres (Fig 6C). Hepatospheres exhibited Cyp1A2 and Cyp3A activity (Fig 6D and 6E) and secreted AFP and albumin over a 4-week period (Fig 6F and 6G). When cultured in 3D, the cell phenotype is more stable and metabolically active (Fig 6D and 6E) compared to previous 2D work [27]. The improved maturation of cells in 3D is evidenced by a significant decrease in AFP secretion over time (Fig 6F). Following their formation, it is common to grow multiple spheroids within a single well, which may impact upon oxygen availability. In order to model this scenario, symmetric properties are neglected and the full 3D model is simulated in COMSOL.

**Fig 7:**
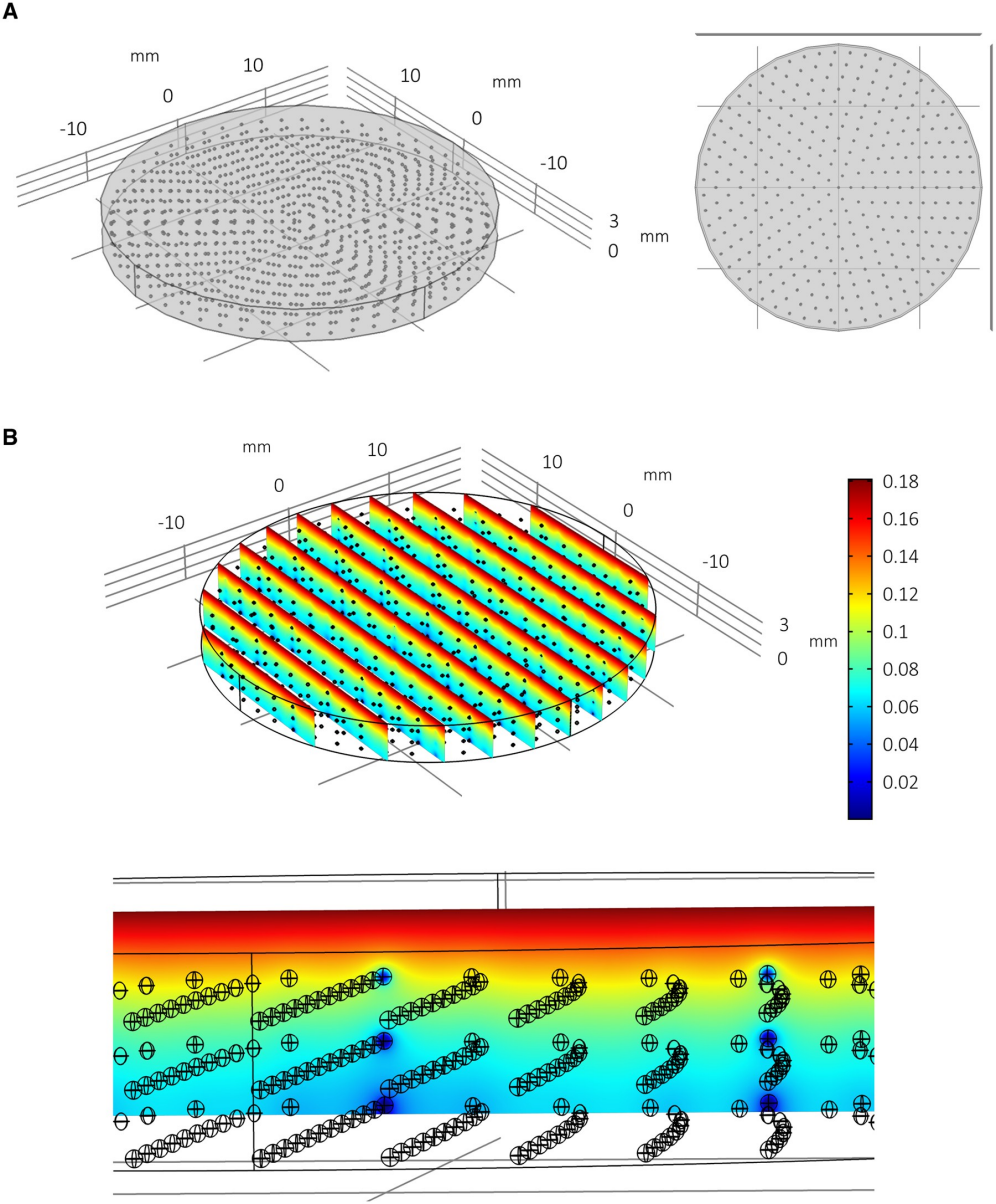
Multiple spheroids per well: Regular array. Model simulation of multiple spheroids per well arranged in a regular array (A) and the consequent impact upon local oxygen concentrations at steady state (B). The radius is fixed at 130 μm for all 993 spheroids. Spheroids towards the bottom of the well have less oxygen.

**Fig 8:**
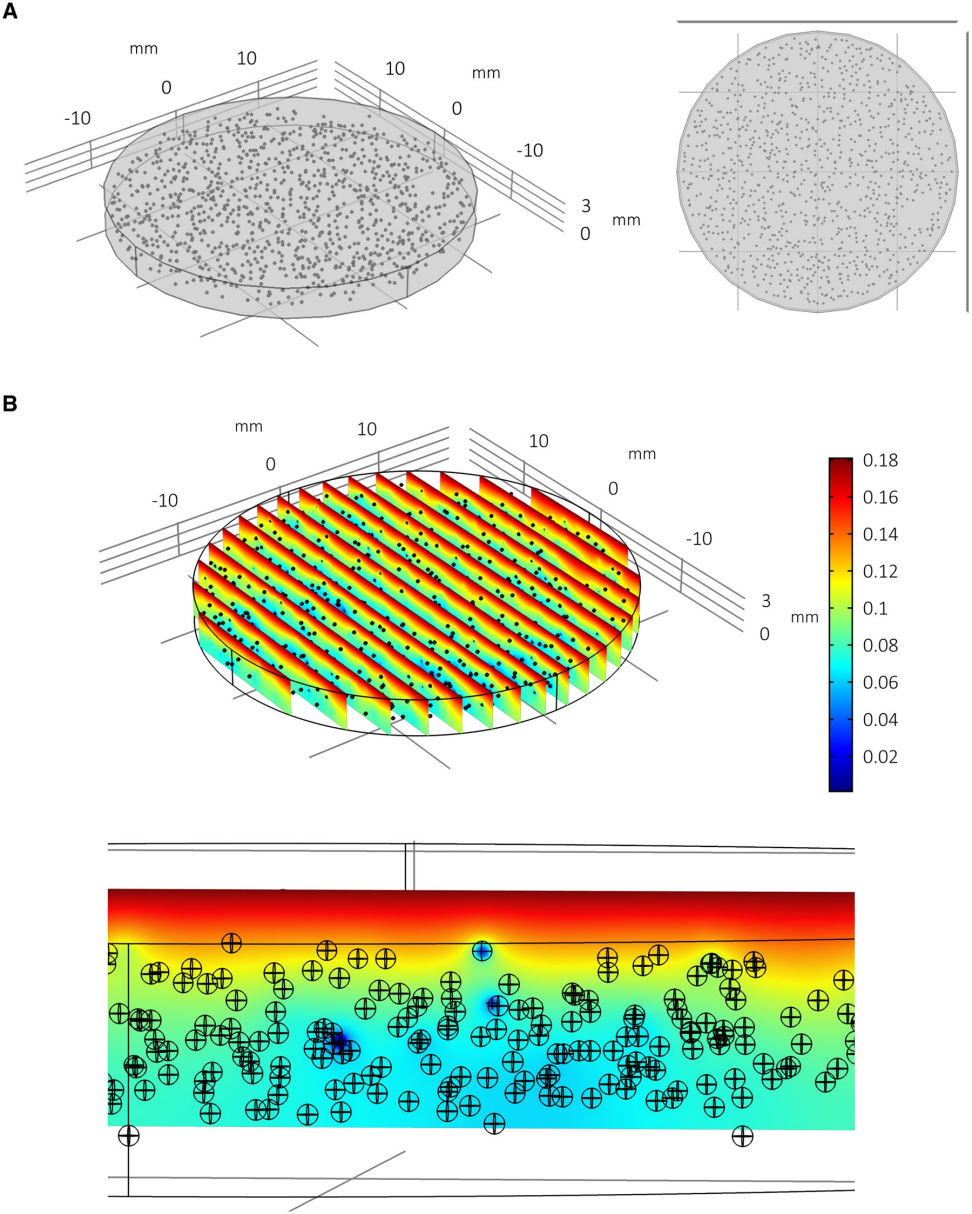
Multiple spheroids per well: Random array. Model simulation of multiple spheroids per well arranged in a randomised array with assumed uniform distribution (A) and the consequent impact upon local oxygen concentrations at steady state (B). The radius is fixed at 130 μm for all 1,000 spheroids. The random distribution of spheroids allows for localised pockets of low oxygen concentrations within the well.

**Fig 9:**
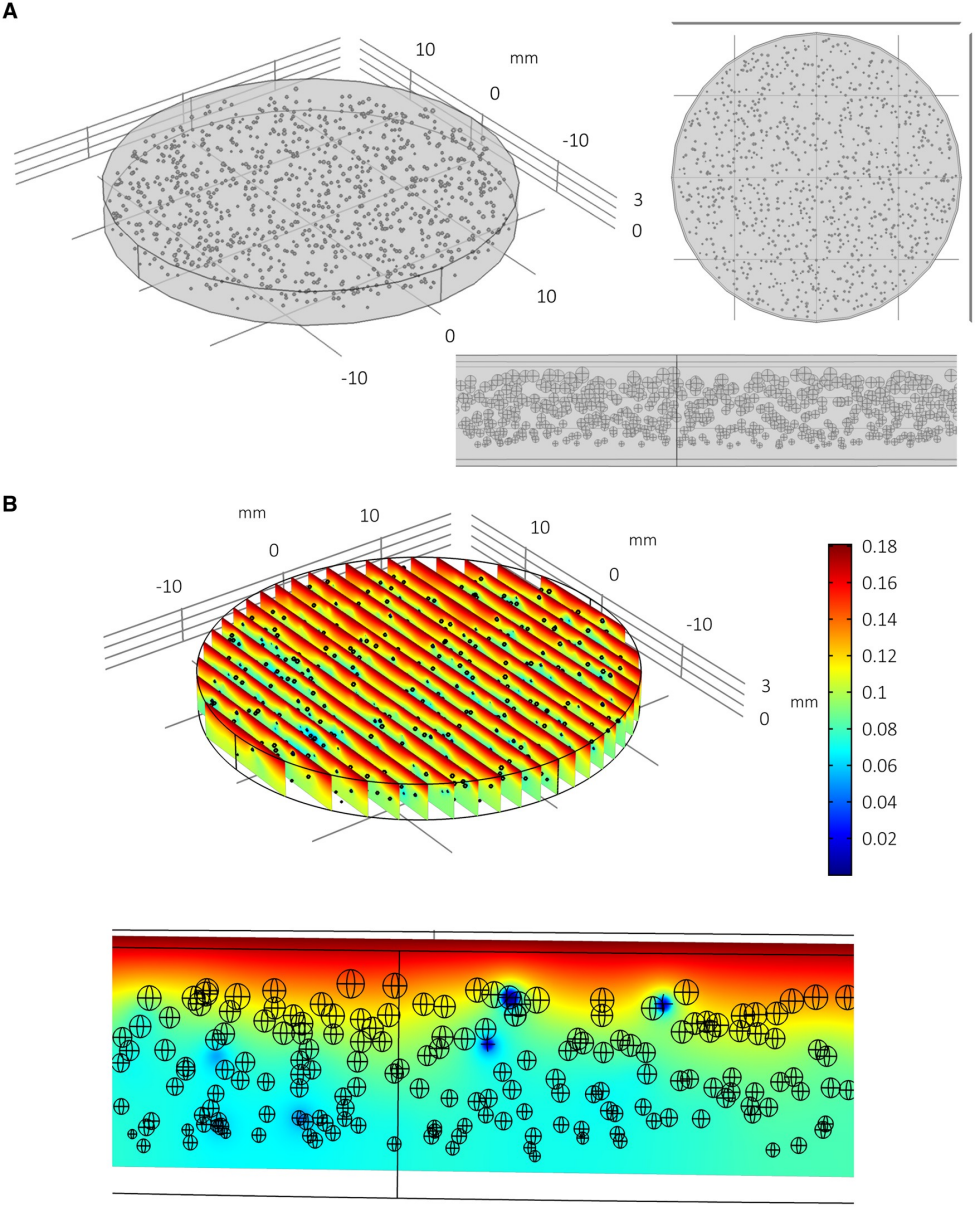
Multiple spheroids per well: Random array with ordered size distribution. Model simulation of multiple spheroids per well arranged in a randomised array with assumed uniform spatial distribution and ordered size distribution (A) and the consequent impact upon local oxygen concentrations at steady state (B). A normal distribution of spheroid radii are assumed (mean 129.71 μm, SD = 22.85 μm) and are positively correlated with spheroid height, i.e. the spheroids at the top are the largest and the spheroids at the bottom are the smallest. This format appears to reduce the overall global depletion of oxygen within the well compared to Figs 7 & 8.

In order to predict the effects of approximately 1,000 spheroids consuming oxygen within this well geometry and media volume, multiple spheroids are generated *in silico* and distributed throughout the well in an array (for an example of the modelling geometry/mesh of multiple spheroids per well, see supplementary Figure S2 in S1 File). Three spheroid arrays are considered: “regular”; “random”; and “random with size variance”. “Regular” spheroid arrays are geometrically idealised distributions consisting of 993 evenly distributed spheroids in 3 stacked circular x-y arrays (see Fig 7A). Each spheroid has radius 130 μm. “Random” arrays consist of 1,000 spheroids (of radius 130 μm) assigned locations randomly within the well such that they do not overlap and are contained within the well geometry (Fig 8A). The “random with size variance” array also consists of 1,000 randomly distributed spheroids. However, their size is determined by their height such that 1,000 radii are drawn from a normal distribution (*r*~*N*(129.72, 22.85^2^)) and assigned to a spheroid in an ordered way such that the largest spheroid corresponds with the highest position in the well (see Fig 9A and supplementary Figure S3 in S1 File). This corresponds with an experimentally observed phenomenon whereby larger PSC-derived liver spheres appear to be located within the upper portion of the media and smaller spheroids within the distribution are found towards the lower portion of the media.

The steady state distribution of oxygen concentration throughout the spheroid arrays and media are plotted in Figs 7B, 8B and 9B with the quantitative metrics summarised in Tables 1 and 2. In Fig 7B it is clear that there is less oxygen available to the spheroids when multiple spheroids are cultured within the same volume of media. Furthermore, spheroids located towards the bottom of the well are relatively hypoxic. The randomised array of spheroids in Fig 8B highlights the potential for localised pockets of hypoxia that may exist within wells where multiple spheroids share the same relatively small amount of space (see heterogeneity in oxygen concentration and dark blue patches). The distribution of spheroid sizes with bigger spheroids positioned towards the upper portion of the media in Fig 9B appears to result in less oxygen depletion within the media (compare colour-coordinated concentrations of Figs 7B and 8B). This calibration of smaller spheroids located towards the bottom and larger spheroids towards the top corresponds with the non-linear nature of the oxygen gradients for these parameters as indicated by the parameter sensitivity analysis conducted for a single spheroid (Fig 3). It follows that larger spheroids are better suited to be positioned towards the oxygen source (media surface) to prevent hypoxia.

**Table 1:** Quantitative oxygen metrics (mmol/L, mmHg and %) for concentrations within the spheroid array.

SPHEROID ARRAY									
	Average O_2_			Maximum O_2_			Minimum O_2_		
	mmol/L	mmHg	~%	mmol/L	mmHg	~%	mmol/L	mmHg	~%
**Regular**	0.036	28	3.68	0.098	75.49	9.91	1.37×10^−4^	0.11	0.014
**Random**	0.041	31.94	4.19	0.118	91.27	12	1.29×10^−4^	0.10	0.013
**Random with size variance**	0.043	33.03	4.34	0.107	82.76	10.9	1.53×10^−7^	1.18×10^−4^	1.6×10^−5^

**Table 2:** Quantitative oxygen metrics (mmol/L, mmHg and %) for concentrations within the media.

MEDIA									
	Average O_2_			Maximum O_2_			Minimum O_2_		
	mmol/L	mmHg	~%	mmol/L	mmHg	~%	mmol/L	mmHg	~%
**Regular**	0.104	80.44	10.56	0.181	140	18.4	0.0052	4.02	0.53
**Random**	0.106	82.00	10.76	0.181	140	18.4	0.0253	19.57	2.57
**Random with size variance**	0.109	84.31	11.07	0.181	140	18.4	0.0373	28.85	3.79

From Tables 1 and 2 it is clear that oxygen levels in the media are very different to intra-spheroidal oxygen levels and therefore should not be used as a proxy measurement. The “random with size variance” is not only the most accurate representation of the observed *in vitro* scenario, but also appears to be the most physiologically relevant for *in vivo* interpretation and extrapolation as the average spheroid concentration appears to be closest to average oxygen concentration within the liver sinusoid. Furthermore, all spheroid arrays predict significant hypoxia in at least some of the spheroids, particularly larger spheroids towards the bottom of the well, due to the number of spheroids and consequent low oxygen supply available locally. The problem of hypoxic media is identified within the regular array but is not found for the randomised arrays (see the minimum oxygen media concentrations).

## 4 Discussion

The *in silico* framework described here was developed by incorporating *in vitro* cell culture information into a mathematical modelling approach. This modelling framework allows for the virtual simulation, investigation and optimisation of experimental conditions in a relatively quick, transparent and economical manner.

The representative results highlight the application of this approach to a novel PSC-derived liver sphere scenario with a tiered modelling system comprising four models (single spheroid within a well; multiple spheroids in regular array; multiple spheroids in randomised array; and multiple spheroids in randomised array with height correlated to size). This stem cell application has vital implications for scaled production of high fidelity and viable liver tissue for further research and transplantation. At each stage of the modelling pathway, from simple to complex tiers, it is possible to gain mechanistic insight into the nature of the system *in vitro*.

By accounting for mechanistic processes within the system explicitly, the researcher can explore the impact of parameters and variables within the system. This can allow for more carefully calibrated experiments and provide more meaningful and physiologically relevant *in vitro* data.

## Supporting information

S1 File (PDF)
