# Supplementary material for: Mathematical modelling of oxygen gradients in stem cell-derived liver tissue
Source: PLoS One. 2021 Feb 8;16(2):e0244070. doi: 10.1371/journal.pone.0244070 (PMC7870006; doi:10.1371/journal.pone.0244070)
Supplement: S1 File — (PDF) [file pone.0244070.s001.pdf]

SUPPLEMENTARY MATERIAL

Mathematical modelling of oxygen gradients in stem cell-derived liver tissue

Joseph A. Leedale[1], Baltasar Lucendo-Villarin[2], Jose Meseguer-Ripolles[2], Alvile Kasarinaite[2], Steven D. Webb[3,4], David C. Hay[2]

[1]Dept. of Mathematical Sciences, University of Liverpool, Liverpool, L69 7ZL, UK
[2]MRC Centre for Regenerative Medicine, University of Edinburgh, Edinburgh, EH16 4UU, UK
[3]Dept. of Applied Mathematics, Liverpool John Moores University, Liverpool, L3 3AF, UK
[4]Current address: Syngenta, Early Stage Research, Product Safety, Jealott's Hill, Bracknell, Berkshire, RG42 6EY, UK

Contents

1 Mathematical model 2
1.1 Model equations 2
1.2 Boundary conditions 2
1.3 Model parameters 2
2 Parameter variation 3
3 COMSOL geometry 4
4 Randomly sampled generation of multiple spheroids within the well 5
References 6

1 Mathematical model

1.1 Model equations

$$\frac{\partial C}{\partial t} = D_{sph}\nabla^2 C - \frac{V_{max}C}{C + K_m}, \quad \text{In the spheroid,} \quad (1)$$

$$\frac{\partial C}{\partial t} = D_{med}\nabla^2 C, \quad \text{In the media.} \quad (2)$$

1.2 Boundary conditions

$$C = C_A, \quad \text{At the air/media interface,} \quad (3)$$

$$\nabla C \cdot \mathbf{n} = 0, \quad \text{At the well/media interface,} \quad (4)$$

$$C_{sph} = C_{med}, \quad \text{At the media/spheroid interface,} \quad (5)$$

$$D_{sph}\nabla C_{sph} = D_{med}\nabla C_{med}, \quad \text{At the media/spheroid interface.} \quad (6)$$

1.3 Model parameters

Model parameter	Value	Reference
D_{sph}	1.60×10^{-9} m^2/s	[1, 2]
D_{med}	4.85×10^{-9} m^2/s	[1, 2]
V_{max}	4.10×10^{-2} $mol/m^3/s$	[3]
K_m	6.24×10^{-3} mol/m^3	[2, 4]

Table 1: Parameter values for the main model.

2 Parameter variation

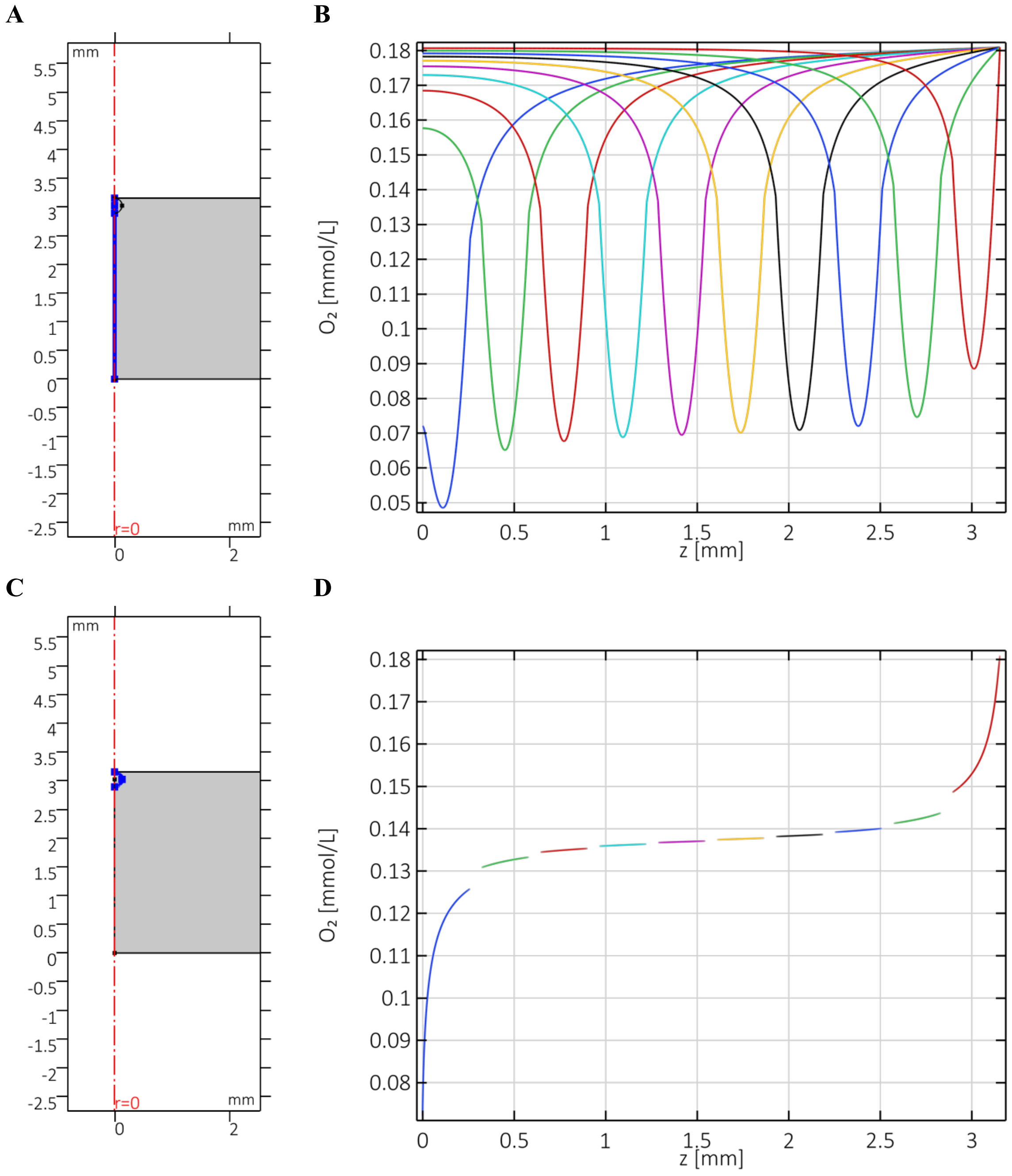

Figure S1: Oxygen profiles along 1D lines either through the central axis of the well (A,B) or around the boundary of the sphere (C,D) for various spheroid heights within the well for a spheroid of radius 130 µm.

3 COMSOL geometry

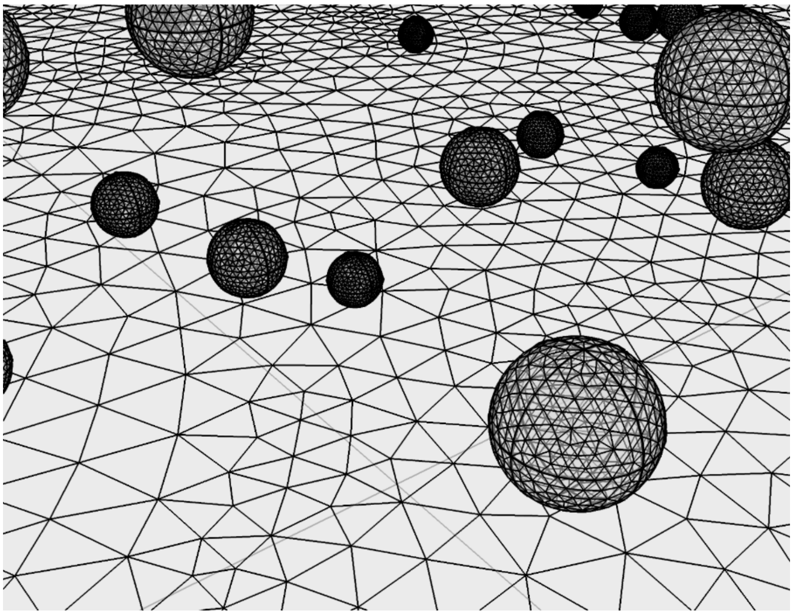

Figure S2: Sample of model geometry in COMSOL with “extremely fine” mesh. Image shows multiple randomly arrayed spheroids within the media.

4 Randomly sampled generation of multiple spheroids within the well

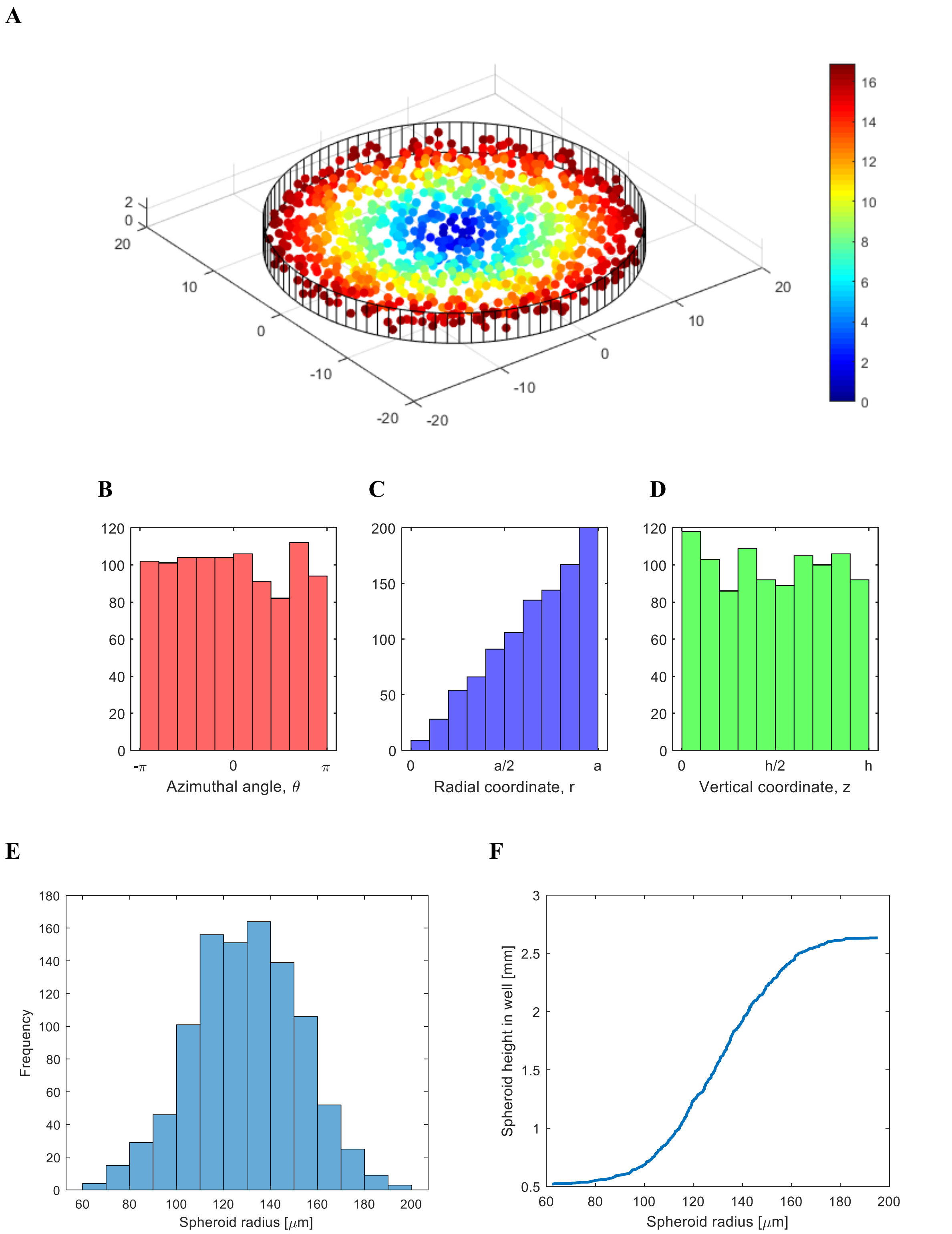

Figure S3: A: 1,000 randomly assigned spheroid coordinates within a virtual well assuming a uniform spatial distribution. Corresponding probability density functions are given for cylindrical coordinates θ (B), r (C), and z (D). E: Randomly generated normal distribution for spheroid radii given mean (129.71 µm) and SD (22.85 µm) measurements. F: Spheroid radii are assigned to each positional coordinate in 3D space based upon the spheroid height (z).

References

1. Leedale, J., et al., *Modeling the dynamics of hypoxia inducible factor-1α (HIF-1α) within single cells and 3D cell culture systems.* Mathematical biosciences, 2014. **258**: p. 33-43.
2. Leedale, J., et al., *In silico-guided optimisation of oxygen gradients in hepatic spheroids.* Computational Toxicology, 2019. **12**.
3. Yu, Y., et al., *Hepatocyte-like cells differentiated from human induced pluripotent stem cells: relevance to cellular therapies.* Stem cell research, 2012. **9**(3): p. 196-207.
4. Shipley, R., et al., *A strategy to determine operating parameters in tissue engineering hollow fiber bioreactors.* Biotechnology and bioengineering, 2011. **108**(6): p. 1450-1461.
